# Dissimilarity of microbial diversity of pond water, shrimp intestine and sediment in Aquamimicry system

**DOI:** 10.1186/s13568-020-01119-y

**Published:** 2020-10-06

**Authors:** Shenzheng Zeng, Sukontorn Khoruamkid, Warinphorn Kongpakdee, Dongdong Wei, Lingfei Yu, Hao Wang, Zhixuan Deng, Shaoping Weng, Zhijian Huang, Jianguo He, Kriengkrai Satapornvanit

**Affiliations:** 1 State Key Laboratory of Biocontrol/Southern Marine Sciences and Engineering Guangdong Laboratory (Zhuhai), School of Marine Sciences, Sun Yat-Sen University, Guangzhou, China; 2 Department of Fisheries, Faculty of Fisheries, Kasetsart University, Bangkok, Thailand; 3 Institute of Aquatic Economic Animals and Guangdong Province Key Laboratory for Aquatic Economic Animals, School of Life Sciences, Sun Yat-Sen University, Guangzhou, China; 4 Guangdong Provincial Key Laboratory of Marine Resources and Coastal Engineering, School of Marine Sciences, Sun Yat-Sen University, Guangzhou, China

**Keywords:** Pacific white shrimp, Aquamimicry system, Microbial community, Water, Sediment, Intestine

## Abstract

The Pacific white shrimp, with the largest production in shrimp industry, has suffered from multiple severe viral and bacterial diseases, which calls for a more reliable and environmentally friendly system to promote shrimp culture. The “Aquamimicry system”, mimicking the nature of aquatic ecosystems for the well-being of aquatic animals, has effectively increased shrimp production and been adapted in many countries. However, the microbial communities in the shrimp intestine and surrounding environment that act as an essential component in Aquamimicry remain largely unknown. In this study, the microbial composition and diversity alteration in shrimp intestine, surrounding water and sediment at different culture stages were investigated by high throughput sequencing of 16S rRNA gene, obtaining 13,562 operational taxonomic units (OTUs). Results showed that the microbial communities in shrimp intestine and surrounding environment were significantly distinct from each other, and 23 distinguished taxa for each habitat were further characterized. The microbial communities differed significantly at different culture stages, confirmed by a great number of OTUs dramatically altered during the culture period. A small part of these altered OTUs were shared between shrimp intestine and surrounding environment, suggesting that the microbial alteration of intestine was not consistent with that of water and sediment. Regarding the high production of Aquamimicry farm used as a case in this study, the dissimilarity between intestinal and surrounding microbiota might be considered as a potential indicator for healthy status of shrimp farming, which provided hints on the appropriate culture practices to improve shrimp production.

## Keypoints

The novel Aquamimicry system has successfully improved shrimp production.

Microbial community in shrimp intestine and environment are investigated.

Relation between intestinal and environmental microbiota indicates culture condition.

## Introduction

Aquaculture remains an important source of food and nutrition for millions of people worldwide, which plays an essential role in meeting the urgent global food demand (Low et al. 2017). Pacific white shrimp, *Litopenaeus vannamei*, with the largest production in global shrimp industry, has suffered enormous economic losses due to serious infectious diseases (Zeng et al. 2020). Diseases caused by viruses and bacteria have been major impact on the sustainable development of the industry, especially the occurrence of white spot syndrome virus (WSSV) (Tassanakajon et al. 2018). Recently, newer diseases have emerged, such as early mortality syndrome (EMS), acute hepatopancreatic necrosis disease (AHPND) or hepatopancreas necrosis syndrome (HPNS) (Flegel 2019; Huang et al. 2016), and white feces syndrome (WFS) (Huang et al. 2020; Zeng et al. 2020). These diseases occurred in many Asian countries including China, India, Indonesia, Thailand and Vietnam. As previously reported, inappropriate aquaculture practices can lead to elevated levels of persistent organic pollutants, metals parasites and viruses in aquaculture field (Amir et al. 2008). Hence, good aquaculture practices should be the main focus of shrimp farming to increase shrimp immunity against diseases and avoid the deterioration of water quality during culture period. Lately, a number of researches have indicated that balance of the microbial community plays a major role in the disease occurrence (Biesebeke 2018; Butto and Haller 2016). Hence, there is a need to develop reliable, repeatable and environmentally friendly technology to combat new and existing pathogens.

The “Aquamimicry System” was established in 2013 by Mr. Sutee Prasertmark and Mr. Veerasan Prayotamornkul, who are long-time shrimp farmers in Thailand. The combination of nature condition and technology leads to more sustainable shrimp farming practices by mimicking the natural aquatic environment. Chemicals used is avoided by employing the symbiotic system, which is created through prebiotics (non-digestion component but can be metabolized by specific microorganism) and probiotics, defined as living microorganisms that have a positive effect on host (Biesebeke 2018; Butto and Haller 2016). The success of this system is based on the production of the natural food for shrimp resulting in the decrease in feed conversion ratio, maintenance of optimal water and sediment quality by microorganisms, and disease elimination (Biesebeke 2018; Butto and Haller 2016). This expertise has been presented to a large number of shrimp producers, and is now adapted in many countries, such as Australia, Bangladesh, Brazil, Brunei, China, Ecuador, Egypt, India, Korea, Malaysia, Mexico, Peru, Singapore, Sri Lanka, USA, and, Vietnam.

As part of this novel shrimp culture technique, the microbial communities of the shrimp intestine and surrounding environment must be studied. Previous studies indicated that shrimp sourced little of their intestinal microbiota from their rearing water, whereas the successions of intestinal microbiota were significantly correlated with host age (Xiong et al. 2019, 2020). Indeed, identification of the microbial community present in the shrimp and nearby environment at different culture stages could facilitate our understanding of the main and important microbial groups which will help to prevent disease outbreaks and maintain good water quality condition during the culture period.

This study investigated the diversity of intestinal microbial community in Pacific white shrimp, sediment, and culture water in a shrimp farm in Nakhon Nayok province, Thailand. This was done by analyzing the types and relationships among the microbial community in sediment, water and shrimp intestines, the relationship of microbial community in shrimp intestine and the surrounding environment, and the microbial alterations at different culture periods. The research results can support the promotion to use of the probiotic and Aquamimicry system in sustainable aquaculture.

## Materials and methods

### Sample collection

The Aquamimicry shrimp farm is located in Nakhon Nayok province, central region of Thailand (14.14^◦^N, 100.99^◦^E). From August to October 2018, samples were collected every 15 days from 15 days post-hatching from four shrimp pond (named as A, B, C and D). Each pond has an area of 4000 m^2^, and 1.5 m depth. Shrimp were cultured at a stocking density ranging from 160,000 to 200,000 shrimps per pond (40–50 shrimp/m^2^). Probiotics were applied during the culture period, such as *Bacillus subtilis, Bacillus amyloliquefaciens, Bacillus valismortis, Bacillus megaterium* and *Bacillus licheniformis*.

Twenty shrimp samples per pond were collected. After measuring length and weight, each shrimp’s surface was separately sterilized with 70% ethanol and the intestine was aseptically dissected into 2 mL sterilized tube containing 1 mL DNA preservation solution.

Water samples were also obtained from three different locations in each pond, with 500 mL water per location. Each water sample was then put in ice separately before filtering through 0.22 μm polyethersulfone filter membranes with a vacuum pump. For the chemical analyses, 200 mL of each sample was collected from the same location using sterile bottles.

Three replicates of sediment samples were collected with 500 mL sterile bottle from different locations. For each sample, 50 g of sediment was used for total nitrogen (TN) and total phosphate (TP) determination, and 10 g of sediment stored into 50 mL sterilized tube for DNA extraction. All the samples were stored at -20 °C before DNA extraction and quality determination.

### Water and sediment quality determination

Water quality parameters such as temperature, pH, dissolved oxygen (DO), and salinity were measured on-site using a YSI handheld multi-parameter instrument (Model YSI 380, YSI Incorporated, Ohio, USA). TN, dissolved inorganic nitrogen (NH_4_^+^-N, NO_2_^−^-N, and NO_3_^−^-N), TP, and orthophosphate (PO_4_^3−^-P) were measured using an automatic discrete analyzer (Model CleverChem 380, DeChem-Tech, Hamburg, Germany). Sediment pH was measured in situ with a pH meter (Model SX630, Sanxin, Shanghai, China).

### DNA extraction and sequencing

Intestine and sediment genomic DNA were extracted by the QIAamp PowerFecal DNA Kit (Qiagen, Dusseldorf, Germany), whereas water DNA was extracted by the MinkaGene Water DNA Kit (mCHIP, Shenzheng, China). The concentration and purity of genomic DNA were measured using the NanoDrop One Spectrophotometer (Thermo Fisher, Massachusetts, USA). The primer pair 515F (5′-GTGCCAGCMG CCGCGGTAA-3′) and 806R (5′-GGACTACHVGGGTWTCTAAT-3′) were used to amplify the V4 region of 16S rRNA gene, which was modified with a barcode tag containing a random 6-base oligos.

PCR products was then purified with EZNA Gel Extraction Kit (Omega, Georgia, USA). Sequencing libraries were generated using NEBNext Ultra DNA Library Prep Kit for Illumina (New England Biolabs, Massachusetts, USA) following manufacturer’s recommendations. The library quality was assessed on the Qubit 2.0 Fluorometer (Thermo Fisher, USA) and Agilent Bioanalyzer 2100 system (Agilent, California, Germany). The libraries were then mixed in equidensity and sent for sequencing by a Hiseq2500 platform (Illumina, California, USA), which was conducted by Guangdong MAGIGENE Biotechnology Co., Ltd. (Guangzhou, China). Raw data generated from Hiseq2500 platform were paired-end reads.

### Bioinformatics and statistical analysis

To control the sequencing quality, raw tags with low quality (quality value < 20, sequence length ≤ 100 bp) were filtered by Trimmomatic (Version 0.33, https://www.usadellab.org/cms/? page = trimmomatic). To merge reads of the same DNA fragment, FLASH (Version 1.2.11, https://ccb.jhu.edu/software/FLASH/) was used to gain the splicing sequences (Magoč and Salzberg 2011). Based on the unique barcode, sequences were assigned to samples and then removed off the barcode and primer sequence by Mothur (Version 1.35.1, https://www.mothur.org) (Schloss et al. 2009).

Sequences with more than 97% similarity were assigned as the same operational taxonomic units (OTU) (Edgar 2013). The chimera sequence and singleton OTU were removed during the OTU clustering by USEARCH (Version 10, https://www.drive5.com/usearch/) (Edgar 2010). For each representative sequence, the SILVA database (Release 132, https://www.arb-silva.de/) was used annotate taxonomic information with the confidence threshold to default to ≥ 0.5. The OTUs abundance information was normalized using a standard of sequence number corresponding to the sample with the least sequences.

Alpha diversity, including Shannon index and Simpson index were calculated with the QIIME (Version 1.9.1, https://qiime.org/) (Caporaso et al. 2010). Bray–Curtis distance was used to evaluate the species complexity differences of samples. Principal coordinate analysis (PCoA) was conducted to reveal the clustering of samples using the vegan package in R (Version 3.6.0). Random forests regression was used to regress relative abundances of taxa in the temporal profiles of water, intestine and sediment samples, using the following parameters with randomForest package in R (cv. fold = 10, step = 0.99, ntree = 5000). To perform the clustering of OTUs for the analysis of time-course data, the fuzzy c-means algorithm was used for the comparative clustering analysis with Mfuzz package in R (Israel et al. 2016). Permutational multivariate analysis of variance (PerMANOVA) was conduct to compare microbial composition dissimilarities (Anderson 2006). A calculated *P* value < 0.05 was considered statistically significant. To evaluate the correlation between environmental factors and microbial community, variation partition analysis (VPA) canonical correlation analysis (CCA) was conducted using the vegan package in R.

## Results

### Shrimp production and environmental factors

The average shrimp length and weight data every 15 days of culture are shown in Additional file 1. Tables S1 and S2. At harvest, the average weights ranged from 23.8 to 25.6 g, production ranged from 3872.6 to 3980 kg/hectare, and survival rates were 95, 100, 82 and 90% for ponds A, B, C and D, respectively. The feed conversion ratio ranged from 1.31 to 1.4, and shrimps were sold at USD 6.38 per kg (Table 1).

**Table 1:** The information of pond production

Details	Pond no			
	A	B	C	D
Pond size (m^2^)	4,000	4,000	4,000	4,000
Culture period (days)	72	72	70	73
Initial stocking	160,000	160,000	200,000	180,000
Stocking density (shrimp/m^2^)	40	40	50	45
Count (shrimp/kg)	39	41	41	42
Harvest size (g)	25.6	24.4	24.4	23.8
Shrimp harvest (kg)	3900.0	3900.0	3980.0	3872.6
Total feed used (kg)	5091.8	5241.8	5053.5	5427.5
Survival rate (%)	95	100	82	90
Feed conversion rate	1.31	1.34	1.27	1.4
Prize (US $/kg)	6.38	6.38	6.38	6.38
Total income (US $)	24,879.3	24,879.3	25,389.7	24,704.5

**Fig. 1:**
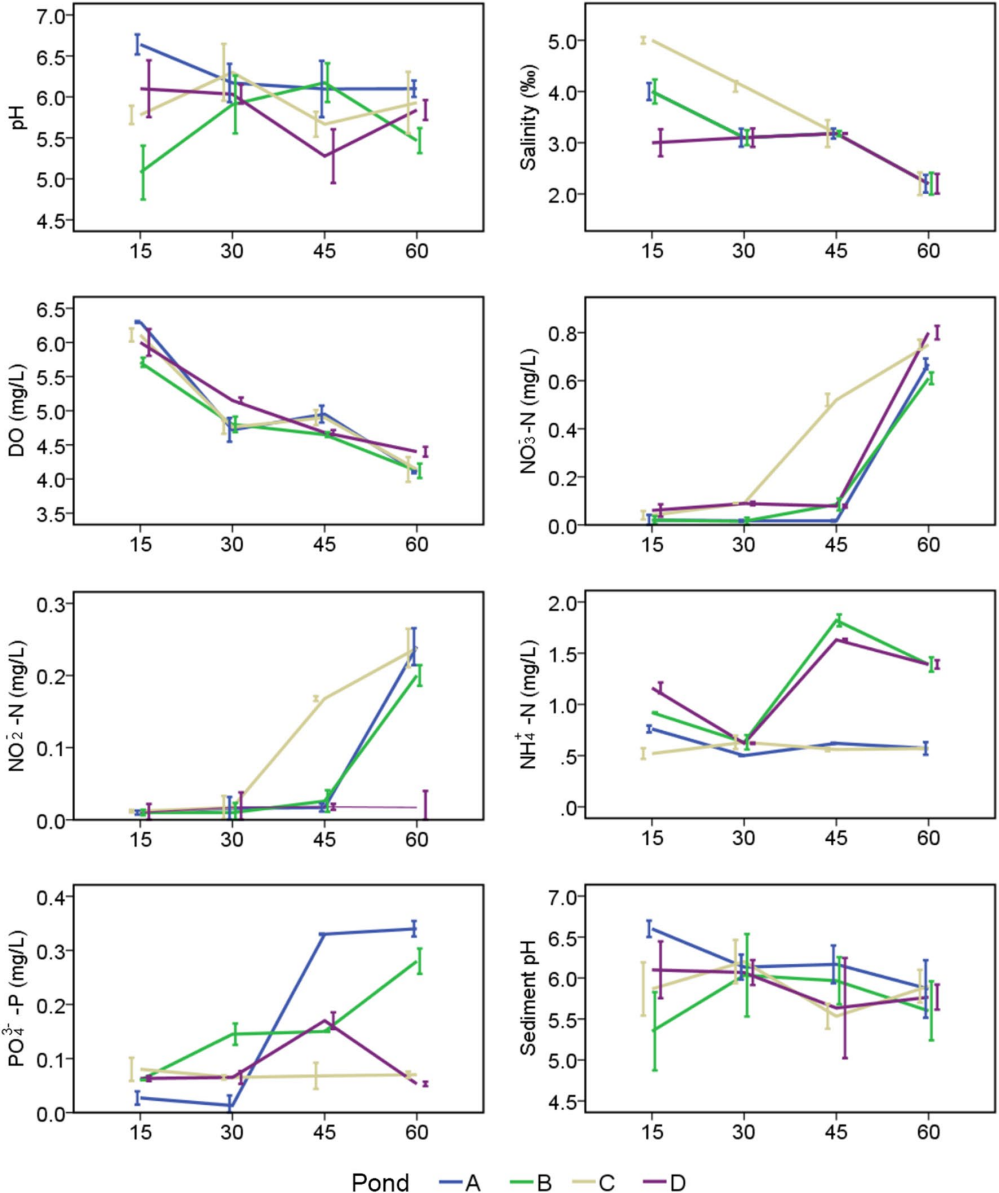
The physicochemical factors of the water and sediment

Water quality parameters were determined throughout the shrimp culture. Temperature was relatively stable at 28–29 °C. Average pH values were between 7.45 and 8.0 (Fig. 1). Weekly salinity gradually decreased during culture from 2–5 (Fig. 1). Likewise, the average dissolved oxygen value decreased from 5.875 mg·L^−1^ to 4.113 mg·L^−1^ (Fig. 1). Average concentrations of NH_3_-N, NO_2_-N, NO_3_-N and PO_4_^3−^ throughout the culture period were between 0.522–1.841 mg·L^−1^, 0.006–0.2516 mg·L^−1^, 0.0117–0.7587 mg·L^−1^ and 0.0359–0.3453 mg·L^−1^, respectively (Fig. 1). Sediment pH value ranged between 5.6 and 6.7 (Fig. 1).

### Microbial composition and diversity in Aquamimicry system

Quality and chimera filtration of the raw data produced a total of 21,657,470 high quality sequencing reads from 162 samples, with an average of 133,688 reads. Finally, 13,562 OTUs were obtained, and the OTUs numbers detected in each sample ranged from 888 to 4,399, with an average of 2190 OTUs. OTUs were identified into 62 phyla and 2203 genera. Sequences that could not be classified into any known groups were assigned as “others”.

In the three habitats, *Proteobacteria*, *Bacteroidetes*, *Planctomycetes*, *Firmicutes*, *Actinobacteria* and *Cyanobacteria* were the dominant phyla. At phylum level, *Proteobacteria* was the most abundant in water (20.8%) and intestine (43.6%), while *Bacteroidetes* was dominant in sediment (32.1%) (Additional file 1. Fig. S1). At the genus level, the most abundant genera were *Exiguobacterium*, *Vibrio*, *Candidatus* Bacilloplasma, *Pirellula*, *Pseudarthrobacter*, *Acinetobacter*, *Rhodopirellula* and *Photobacterium* (Additional file 1. Fig. S2).

**Fig. 2:**
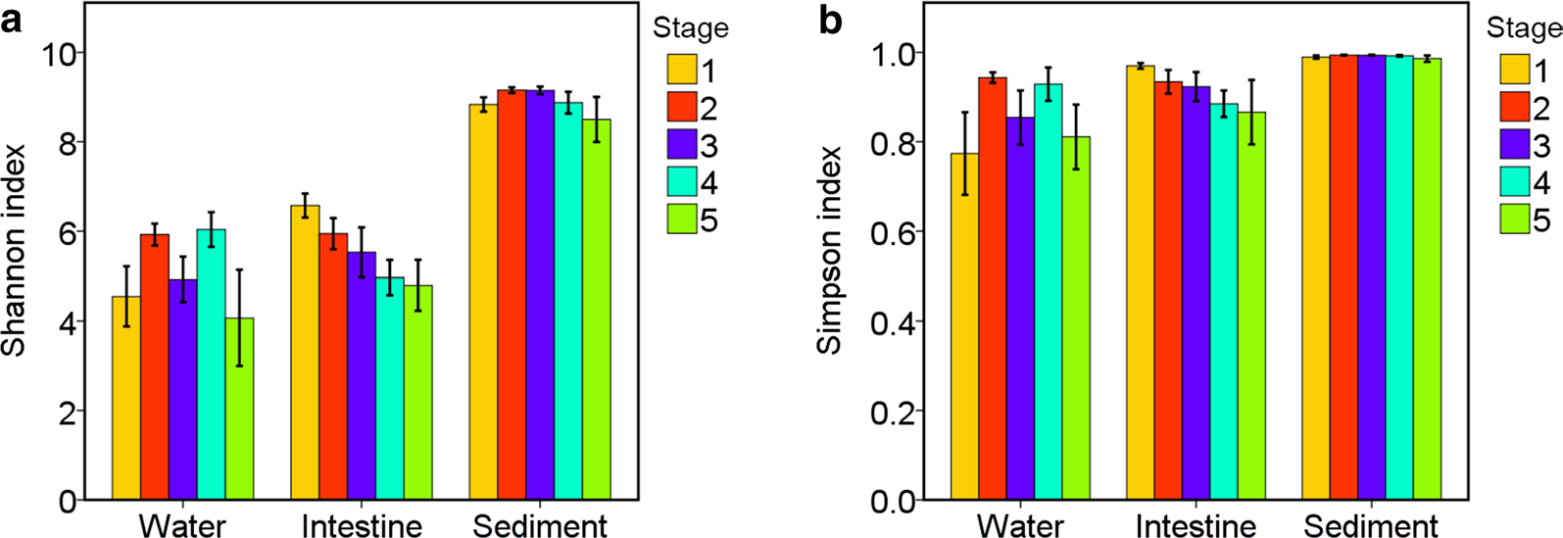
The *α*-diversity comparison among three habitats. **a** The Shannon index **b** The Chao1 index. Compared to water and intestine samples, the *α*-diversity of sediment microbiota was the highest

To estimate and compare the bacterial diversity in the three habitats, α-diversity indices (Chao1 and Shannon index) were calculated from OTUs of each sample. The Shannon indices of water, intestine and sediment were 5.2 ± 1.0, 5.6 ± 0.8 and 8.9 ± 0.3 respectively (Fig. 2a). The Chao1 indices of water, intestine and sediment were 1,673 ± 495, 1,320 ± 336 and 4,038 ± 287 respectively (Fig. 2b), which suggested that the sediment microbial community held the highest α-diversity.

### Microbial similarities and differences among the three habitats

**Fig. 3:**
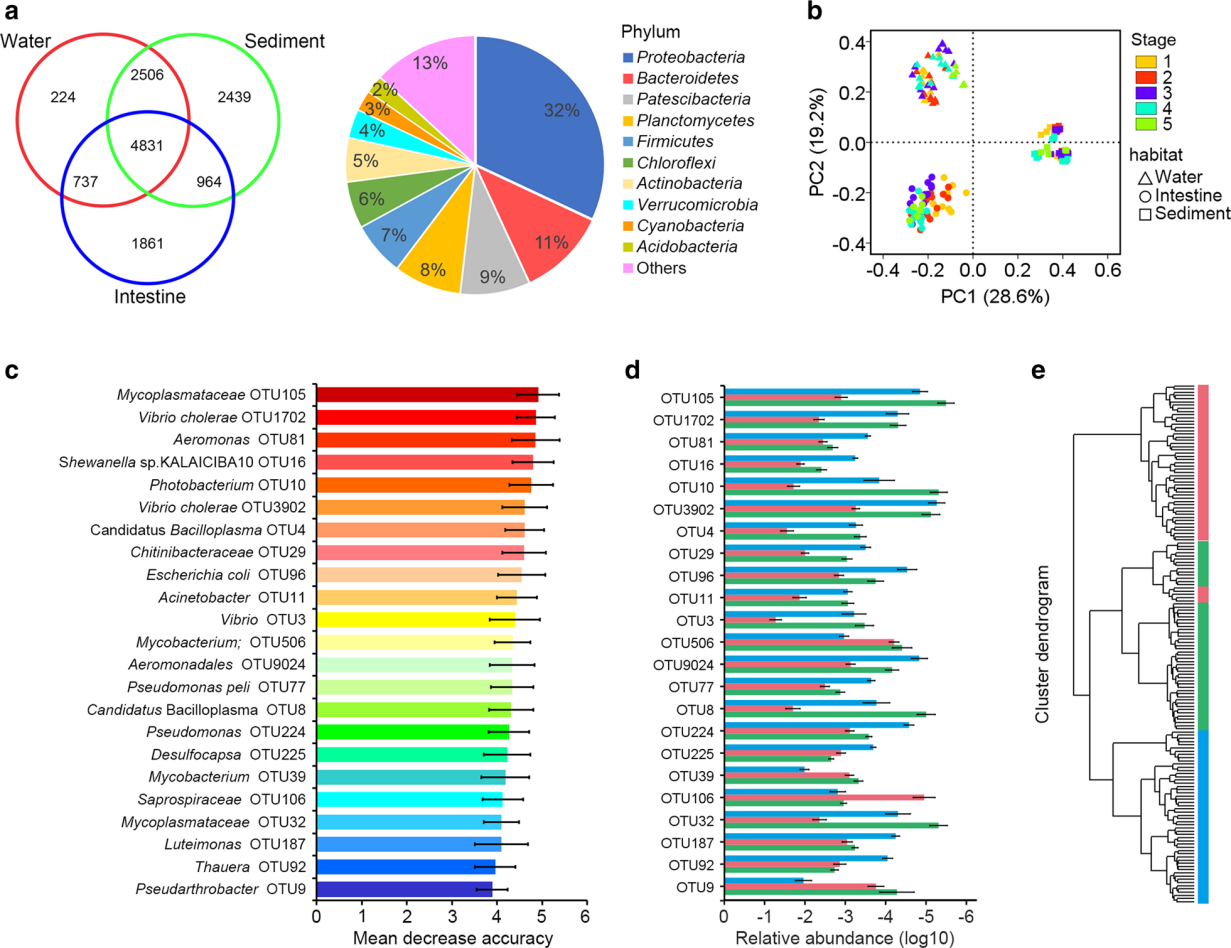
The relationship between shrimp intestinal and environmental microbiota. **a** Venn diagram shows the unique and shared OTUs in the different habitats. the shared OTUs majorly belonged to *Proteobacteria* and *Bacteroidetes* phylum. **b** PCoA of microbial communities based on the 16S rRNA sequencing profiles. Samples were clustered into three groups by PCoA based on Bray–Curtis distance, indicating that the microbial structure differed significantly among three habitats. **c** Classification of the OTU markers for the three habitats. A total of 23 OTU markers were selected as the optimal marker set by a ten-fold CV-error curve. **d** The relative abundance of the OTU markers at each group. **e** UPGMA clustering based on the 23 markers revealed that the microbial composition of each group was clearly distinct

For further investigation of the dominant microbiota that exists in the three habitats, a Venn diagram revealed that 4,831 OTUs were shared in shrimp intestine, surrounding water and sediment (Fig. 3a). These OTUs hold a large percentage of the total bacterial population (average of 81.5, 85.7 and 76.5% in water, intestine and sediment, respectively). In addition, unique OTUs in three habitats were observed, such as 224, 1,861, 2,439 unique OTUs were only found in water, intestine and sediment, respectively. PCoA analysis revealed that the microbial communities of the three habitats were markedly distinct from each other (Fig. 3b).

To find out the specific bacterial taxa distributed among the three habitats, we constructed a random forest classifier model that could specifically identify samples in each group. To detect unique OTU markers, a ten-fold cross-validation on a random forest model with all 162 samples was conducted. Results indicated that a total of 23 OTU markers were selected as the optimal marker set. The relative abundance of these OTUs in each group was presented (Fig. 3c), which were further proven to be significantly different among the three habitats by ANOVA (*P* < 0.05) (Fig. 3d). Compared to the surrounding water and sediment, some opportunistic pathogens (*Vibrio*, *Aeromonas*, *Photobacterium* and *Candidatus* Bacilloplasma) were significantly sufficient in the intestine (*P* < 0.05). The UPGMA clustering showed that almost all the individual samples were clustered into groups according to habitat (Fig. 3e), which suggested that the 23 OTU markers were a successful in distinguishing the specific taxa in each habitat.

### Microbial alterations during different culture periods

**Fig. 4:**
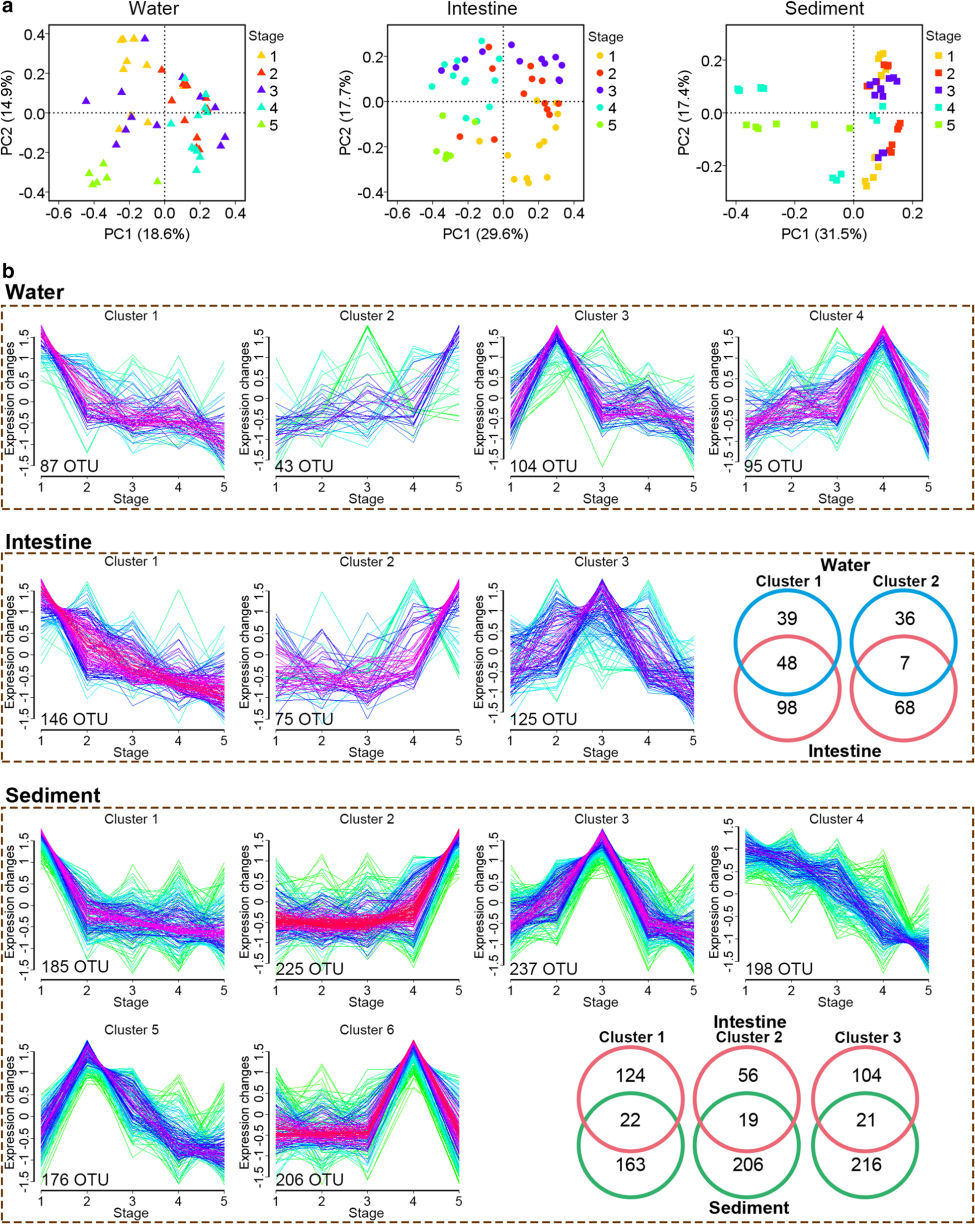
The altered microbial structure at different culture stages. **a** PCoA of the microbial structure at different culture stages. **b** OTUs with similar abundance alteration at different stages clustered into different groups. All the OTUs that were presented in more than 50% samples at each habitat were used for Mfuzz analysis. The x-axis represents culture stage, and the y-axis represents standardized expression change. OTUs in the same cluster suggested that their alteration trends during the culture period were similar. The Venn diagram showed the shared OTU number between similar clusters

The difference in microbial communities at different culture stages was further investigated. In the three habitats, PCoA results showed that all the individual samples were clustered into groups according to stage of culture (Fig. 4a). PERMANOVA (*P* < 0.05) confirmed that the microbial communities differed significantly between any two of the compared stages (*P* < 0.05), except between stages 2 and 3 (*P* > 0.05) (Table 2).

**Table 2:** PERMANOVA of microbial structure at different stages

Habitat	Stage	1	2	3	4	5	R^2^
Water	1	–					0.215
	2	0.01**	–				
	3	0.03*	0.18	–			
	4	0.01**	0.03*	0.12	–		
	5	0.01**	0.01**	0.01**	0.01**	–	
Intestine	1	–					0.278
	2	0.01**	–				
	3	0.01**	0.1	–			
	4	0.01**	0.01**	0.02*	–		
	5	0.01**	0.01**	0.01**	0.01**	–	
Sediment	1	–					0.337
	2	0.53	–				
	3	0.06	0.41	–			
	4	0.01**	0.01**	0.01**	–		
	5	0.01**	0.01**	0.01**	0.02*	–	

PERMANOVA was used to compare significant differences at different culture stages

Significant differences are indicated by asterisks (*, *P* < 0.05; **, *P* < 0.01)

For further determination of which variables affected the shrimp intestinal microbial structure, a VPA was conducted. A sunset of environmental parameters explained 9.0% of the observed microbial variation, while the effect of different culture stages was more pronounced (11.2%) (Additional file 1. Fig. S3a). CCA was applied to reveal the correlation between environmental parameters and microbial community (Additional file 1. Fig. S3b). Salinity, water pH and DO were correlated to microbial community, which indicated that they were the crucial factors shaping the variations in microbial community.

To identify the altered taxa at different stages and how the relative abundance altered, a comparative clustering strategy was implemented to detect substantial differences in abundance profiles across stages. The fuzzy c-means clustering grouped similar abundance profiles into 3, 4 and 6 clusters in water, intestine and sediment, respectively, representing distinct phases of abundance during the culture period (Fig. 4b). This strategy allowed us to easily identify which taxa exhibited the same abundance patterns. Results showed that a great amount of OTUs (water: 328; sediment: 1228; intestine: 346) exhibited a dramatic alteration during the culture period, which corresponded to the results of PCoA and PERMANOVA analysis that microbial composition varied significantly at different culture stages. Moreover, the shared OTUs in the same alteration pattern was observed. There were 22 (15%), 19 (25%) and 21 (17%) shared OTUs between intestine and sediment in clusters 1 to 3, while 48 (33%) and 7 (0.9%) shared OTUs were found between intestine and water (Fig. 4b), which indicated that the intestinal microbial alteration did not correlated much to water and sediment during the culture period.

## Discussion

A number of studies suggest that shrimp intestinal microbiota is closely related to rearing environmental microbiota, which is also linked to shrimp disease occurrence (Hou et al. 2018b; Xiong et al. 2018a). The Aquamimicry system provides an ecological pattern to prevent disease outbreaks in shrimp culture. However, little is known about the microbial composition and diversity alterations of this system in shrimp intestine and the surrounding environment. In this study, the relationship of microbial community between shrimp intestine and the surrounding environment was investigated, and the altered microbes during the culture period were further identified. This work partially meets the urgent need for understanding the main and important microbial community in Aquamimicry system.

Many studies report that the interactions of intestinal microbiota and surroundings were associated with aquatic animal diseases (Li et al. 2017b; Xiong et al. 2018a). Thus, observation of the overlap between shrimp intestinal microbiota and that of their surrounding water and sediment is necessary for shrimp health management in aquaculture. A previous study on shrimp earthen ponds demonstrated that similar bacterial community compositions were observed in shrimp intestine, the surrounding water and sediment (Hou et al. 2018b). Moreover, some reports have shown similar microbial community in intestine and surroundings of crucian carp (Li, et al. 2017a). However, unlike in these previous researches, this study revealed that microbial communities of the three habitats were markedly separate (Fig. 3). Furthermore, the shared OTU numbers were relatively low between the same alteration pattern of intestinal microbiota and surrounding microbiota (Fig. 4), which indicated that the microbial community in shrimp intestine was not the same as those in the surrounding water and sediment in the Aquamimicry system. A previous study exhibited similar changes between water and intestinal microbiota during the culture period, suggesting that the water microbiota altered the shrimp intestinal microbiota, and was subsequently related to disease outbreak (Xiong, et al. 2018a). The same phenomenon was found in crucial carp suffered from red-operculum disease that a close link between intestinal and environmental microbiota (Li, et al. 2017a). Similar results were found in a tilapia farm that pond water and sediment bacteria influenced the composition of intestine, especially the presence of pathogens, such as *Streptococcus* and *Vibrio* (Al-Harbi and Uddin 2005). Further study demonstrated that tilapia suffered from infections caused by an emerging *Francisella* sp. which was found in sediment and water with high abundance (Soto, et al. 2009). As the Pacific white shrimp is a typical benthos that takes in feed debris or sediment from the pond bottom, some culture operations in the Aquamimicry system, such as removal the feed from the bottom and using multiple oxygen increasing device to agitate the water thoroughly, can effectively reduce shrimp’s intake of debris, which might weaken the effect of the environmental harmful factors on shrimp. As previously reported, shrimp sourced little of their intestinal microbiota from their rearing water, whereas the successions of intestinal microbiota were significantly correlated with host age (Xiong et al. 2019, 2020). In the present study, the culture stages were more pronounced than environmental factors in microbial variations (Additional file 1. Fig. S3), while the shared OTUs at the same stages were low (Fig. 4), which indicated that both culture stages and aquaculture pattern affected shrimp intestinal microbial composition. Taken all above clues into consideration, the divergence between shrimp intestinal and environmental microbiota in the Aquamimicry system may be a potential indicator for the healthy condition of shrimp culture, which give us inspiration on the sustainable shrimp culture practice.

Microbial diversity, particularly, intestinal diversity, may be considered a feature of host health (Ren et al. 2018). In this study, the α-diversity (Shannon index and Chao1 index) deceased during the culture period (Fig. 2). In a previous study, shrimp intestinal microbial diversity altered significantly at different culture stages (Zeng et al. 2017). Similar results have been observed in the intestinal microbiota of aquaculture fishes where the α-diversity decreases over culture period (Yan et al. 2016). Another study suggested that larval shrimp can obtain sufficient and diverse bacterial species rapidly from the environment, although only a little part of the microbes could successfully colonize in the shrimp intestine (Xiong et al. 2018a). Some studies reveal that the development of animal digestive and immune systems may significantly affect the intestinal microbiota (Costello et al. 2012; Yan et al. 2016). The larvae, without complete immunity, may easily obtain more species from the environment, whereas the adult shrimp can selectively acquire different types of microbes. This finding is consistent with the processes found in other aquatic animals wherein microbial diversity decreases with host development (Burns et al. 2016; Yan et al. 2016). This information could be important for further microbial management for a more beneficial intestinal microbiota in shrimp culture, which might assist shrimp in preventing opportunistic pathogen or disease outbreaks.

Many investigations demonstrated that the important roles of intestinal microbial composition in aquatic animal health (Hou et al. 2018a; Li et al. 2017a, 2016; Xiong et al. 2018b). In addition, the intestine may host opportunistic pathogens, and it is evidenced that the overgrowth of opportunistic pathogens could contribute to disease (Perez et al. 2010; Xing et al. 2013). For example, in our previous study, the overgrowth of *Candidatus* Bacilloplasma and *Phascolarctobacterium* may contribute to white feces syndrome (Hou et al. 2018a). Similarly, *Vibrio*, *Aeromonas* and *Photobacterium* are generally known to include main opportunistic bacteria that could be pathogenic to aquatic animals (Nayak 2010; Zhang et al. 2014). Herein, the abundance of some opportunistic pathogens was relatively low (*Aeromonas*, 0.5; 10.7%; *Phascolarctobacterium*, < 0.01%; *Photobacterium*, 0.4%;), whereas *Vibrio* (10.9%) and *Candidatus* Bacilloplasma (10.4%) were the dominant genera in shrimp intestine. This phenomenon was also detected in another study (Li et al. 2017a), which suggested that these genera may act not only as opportunistic pathogens, but also as essential players with other unknown functions in the intestine environment. In addition, some probiotics were added during the culture period. However, the relative abundance of *Bacillus* was low in the three habitats (< 0.001%), which indicated that the use of probiotics did not effectively establish a large population in intestine and environment as expected. Unlike *Bacillus*, another probiotic *Lactococcus* was higher than 0.1% proportion in shrimp intestine. These results provide valuable clues for guiding the rational use of probiotics in shrimp culture.

Collectively, the composition and diversity of shrimp intestine microbiota and surrounding microbial community in Aquamimicry system were evaluated, with further investigation on the relationship between intestinal microbiota and the surrounding water and sediment. The microbial diversity of intestine and surroundings was different in Aquamimicry system, which indicates that dissimilarity among the three habitats could be a potential indictor for the healthy situation of shrimp culture, and will give us guidance for the sustainable shrimp culture practice to effectively promote the shrimp production. These findings may strengthen our understanding of the significance of microbial community in Aquamimicry system, and offer fundamental information to improve healthy culture practices in shrimp farming.

## Supplementary information

**Additional file 1:** **Table S1. **Average shrimp weight performance every 15 days (g). **Table S2. **Average shrimp length performance every 15 days (cm). **Figure S1. **Microbial composition of water, intestine and sediment habitats at phylum level. **Figure S2. **Microbial composition of water, intestine and sediment habitats at genus level. **Figure S3. **Effect of culture stages and water physical chemical factors on microbial community.

## Data Availability

The 16S rRNA gene sequencing data and metagenomic data used in this study are available in the NCBI Short Read Archive (https://www.ncbi.nlm.nih.gov/sra) under Bioproject PRJNA625419.
