# Supplementary material for: Dissimilarity of microbial diversity of pond water, shrimp intestine and sediment in Aquamimicry system
Source: AMB Express. 2020 Oct 6;10:180. doi: 10.1186/s13568-020-01119-y (PMC7538476; doi:10.1186/s13568-020-01119-y)

AMB Express

Dissimilarity of microbial diversity of pond water, shrimp intestine and sediment in Aquamimicry system

Shenzheng Zeng[1*], Sukontorn Khoruamkid[2*], Warinphorn Kongpakdee[2], Dongdong Wei[1,3], Lingfei Yu[1], Hao Wang[1,3], Zhixuan Deng[1], Shaoping Weng[1,3,4], Zhijian Huang[1,3,4,★], Jianguo He[1,3,4,★], Kriengkrai Satapornvanit[2,★]

1 State Key Laboratory of Biocontrol / Southern Marine Sciences and Engineering Guangdong Laboratory (Zhuhai), School of Marine Sciences, Sun Yat-sen University, Guangzhou, China

2 Department of Fisheries, Faculty of Fisheries, Kasetsart University, Thailand

3 Institute of Aquatic Economic Animals and Guangdong Province Key Laboratory for Aquatic Economic Animals, School of Life Sciences, Sun Yat-sen University, Guangzhou, China

4 Guangdong Provincial Key Laboratory of Marine Resources and Coastal Engineering, School of Marine Sciences, Sun Yat-sen University, Guangzhou, China

*These authors contributed equally to this work

★Corresponding Authors

Zhijian Huang

Email: lsshzhj@mail.sysu.edu.cn; Phone: +86 13533553570; Fax: 020-84113793

Jianguo He

Email: lsshjg@mail.sysu.edu.cn; Phone: +86 13825090465; Fax: 020-84113793

Kriengkrai Satapornvanit

Email: ffiskks@ku.ac.th; Phone: +66 (0)86 812 9822; Fax: +662942-8364

Supplementary Table S1 Average shrimp weight performance every 15 days (g)

Pond no.	Days of culture			
	15	30	45	60
A	0.73±0.02	1.68±0.31	10.37±1.06	20.62±1.57
B	0.75±0.03	1.60±0.16	11.49±0.83	21.46±1.34
C	0.58±0.12	6.92±0.78	14.47±1.33	20.60±1.52
D	0.69±0.12	6.73±0.76	14.48±1.14	20.35±1.66

Supplementary Table S2 Average shrimp length performance every 15 days (cm)

Pond no.	Days of culture			
	15	30	45	60
A	5.19±0.15	8.59±0.48	11.20±0.38	13.73±0.31
B	5.32±0.24	8.51±0.35	11.50±0.27	13.71±0.29
C	5.94±0.33	9.7±0.41	11.9±0.39	13.66±0.43
D	6.37±0.35	9.52±0.44	12.25±0.40	13.79±0.25

Supplementary Fig. S1 Microbial composition of water, intestine and sediment habitats at phylum level. The relative read abundance of different bacterial phyla within the different communities.

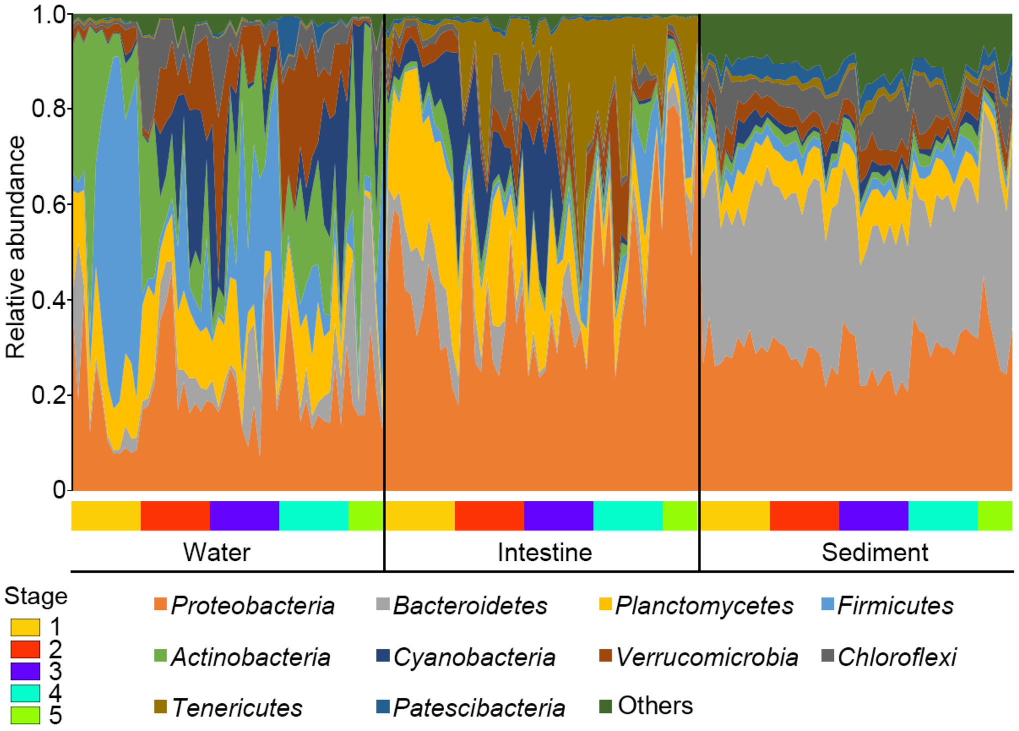

Supplementary Fig. S2 Microbial composition of water, intestine and sediment habitats at genus level. The relative abundance of each column was normalized to Z score in heatmap.

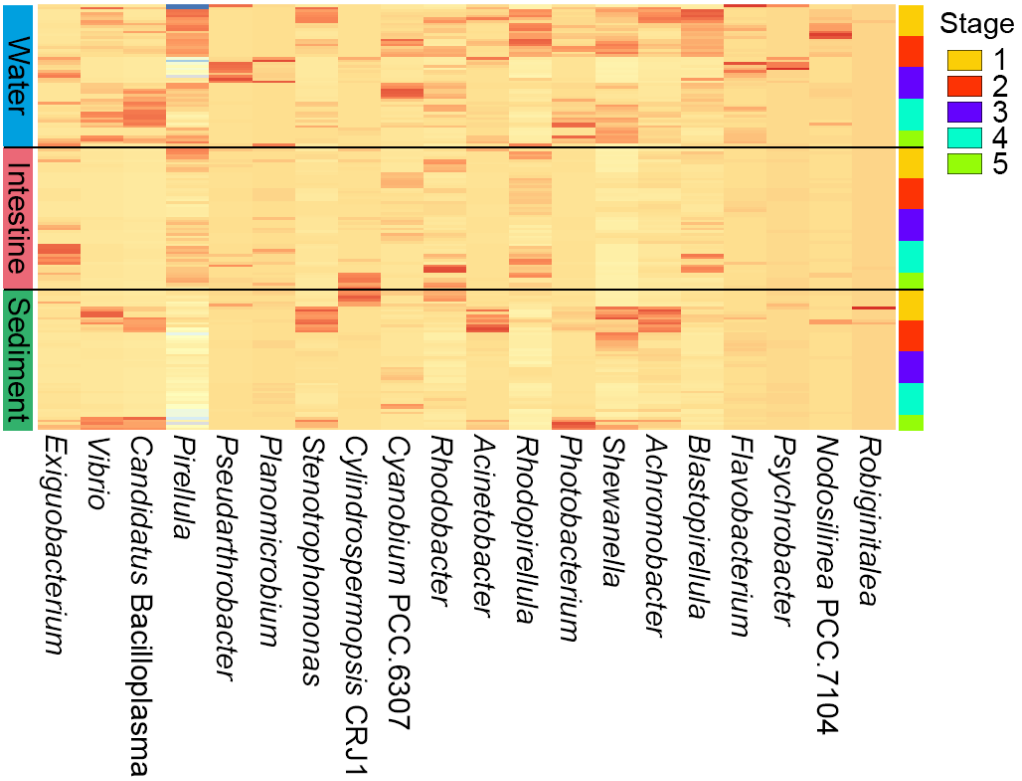

Supplementary Fig. S3 Effect of culture stages and water physical chemical factors on microbial community. (a) Variation partition analysis (VPA) of the effects of culture stages and environmental parameters on the microbial structure of shrimp intestinal communities. (b) Effects of environmental parameters on sample separation using canonical correlation analysis (CCA).

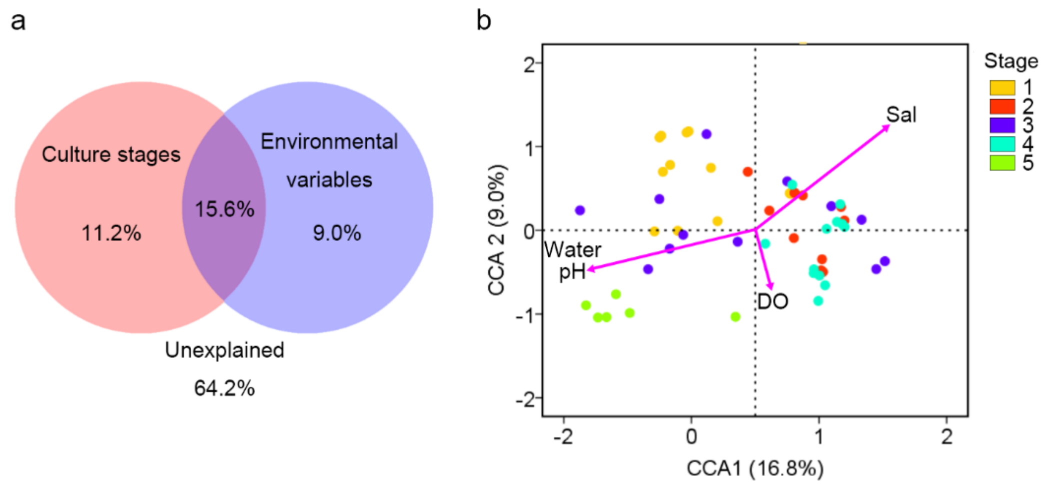

Supplement: Supplementary file 1 — Additional file 1: Table S1. Average shrimp weight performance every 15 days (g). Table S2. Average shrimp length performance every 15 days (cm). Figure S1. Microbial composition of water, intestine and sediment habitats at phylum level. Figure S2. Microbial composition of water, intestine and sediment habitats at genus level. Figure S3. Effect of culture stages and water physical chemical factors on microbial community. [file 13568_2020_1119_MOESM1_ESM.pdf]
